# Combining Dedicated Online Training and Apprenticeships in the Field to Assist in Professionalization of Humanitarian Aid Workers: a 2-year Pilot Project for Anesthesia and Intensive Care Residents Working in Resource Constrained and Low-income Countries

**DOI:** 10.1371/currents.dis.4b85b2e37f58297619faac3141f0d3e3

**Published:** 2014-07-21

**Authors:** Marco Foletti, Pier Luigi Ingrassia, Luca Ragazzoni, Ahmadreza Djalali, Alba Ripoll Gallardo, Frederick M. Burkle, Francesco Della Corte

**Affiliations:** Center for Research and Education in Emergency and Disaster Medicine, Department of Translational Medicine, University of Eastern Piedmont, Novara, Italy; Center for Research and Education in Emergency and Disaster Medicine, Department of Experimental and Clinical Science, University of Eastern Piedmont, Novara, Italy; Research Center in Emergency and Disaster Medicine and Computer Science Applied to Medical Practice (CRIMEDIM), Department of Translational Medicine, Università degli Studi del Piemonte Orientale “A. Avogadro”, Novara, Italy; Center for Research and Education in Emergency and Disaster Medicine, University of Eastern Piedmont, Novara, Italy; Research Center in Emergency and Disaster Medicine and Computer Science Applied to Medical Practice (CRIMEDIM), Department of Anesthesia and Critical Care, Novara, Italy; Harvard Humanitarian Initiative, Harvard School of Public Health, Harvard University, Cambridge, Massachusetts, USA; Center for Research and Education in Emergency and Disaster Medicine, Department of Experimental and Clinical Science, Novara, Italy. Academy for Emergency Management and Disaster Medicine (EMDM Academy).

## Abstract

Introduction: As a result of the gaps in humanitarian response highlighted by several reports, the international community called for an increased professionalization of humanitarian aid workers. This paper describes a pilot project by an Italian university and a non-profit, non-governmental organization to implement a medical apprenticeship in low-income countries during Anesthesia and Intensive Care Medicine residencies. Methods: Before deployment, participants were required to complete a dedicated online training course about safety and security in the field, principles of anesthesia at the district hospital level, emergency and essential surgical care, essentials of medical treatment in resource-constrained environments and psychological support in emergencies. Results: At the end of the program, a qualitative self-evaluation questionnaire administered to participants highlighted how the project allowed the participants to advance their professional skills when working in a low-resource environment, while also mastering their adapting skills and the ability to interact and cooperate with local healthcare personnel. The project also proved to be a means for personal growth, making these experiences a recommendation for all residents as a necessary step for the professionalization of healthcare personnel involved in humanitarian aid.

## Introduction

In 2010 there was an estimated total 274,000 humanitarian workers worldwide with a growth rate of 4% from the previous year. Although the system is still dominated, in terms of operational presence and resource share, by a small group of NGOs and international organizations whose combined humanitarian expenditure in 2010 exceeded $2.7 billion, there are an estimated 4,400 NGOs worldwide that undertake humanitarian action on an ongoing basis^1^ .

This heterogeneous and fragmented nature of the humanitarian sector has hindered its ability to efficiently respond to crises worldwide^2^ , and concerns have been expressed on the clinical competencies and practices by foreign medical teams (FMTs) during response to large scale disasters such as the 2010 Haiti earthquake or the 2004 South Asian tsunami^3^ . This resulted in the international community calling for increased professionalization of the humanitarian sector through “an international professional association, the development of core competencies that drives curricula, and the creation of a universal certification system for aid workers”^4^ . A survey conducted among professionals involved in humanitarian assistance further confirmed those needs^5^ . In this respect, the Consortium of British Humanitarian Agencies (CBHA) published a framework for core and leadership humanitarian competencies^6^ that has served as a model for developing education and training curricula.

However, as highlighted by the U.K. Department for International Development (DFID) Humanitarian Emergency Response Review, there is still “no straightforward professional route into humanitarian work” and “in every major emergency there are still significant numbers of aid personnel who lack some of the skills essential to their jobs”^7^ . Earlier programs to promote global health among residents found many barriers to the successful implementation in the academic curriculum, such as time constraints, lack of approval and funding concerns^8^ .

Due to these factors, the Research Center In Emergency and Disaster Medicine and Computer Science Applied To Medical Practice (CRIMEDIM)^9^ in collaboration with Rainbow for Africa Onlus^10^ , an Italian non-profit organization, decided to design and implement a pilot training course for residents in Anesthesia and Intensive Care Medicine. This combined theoretical and essential knowledge and an onsite tutored internship in a resource-constrained field simulation as an internationally recommended apprenticeship. The ultimate goal was to better prepare future humanitarian aid workers through a standard professionalization process.

This paper reports on the 2-years pilot project detailing the characteristics and features of the training course and the apprenticeship as well as the feedback of the participants.

**Institutions Involved**

CRIMEDIM is an interdepartmental university research center established in 2007 at the Università of Piemonte Orientale (Novara, Italy) devoted to the medical scientific study of disasters. Its aims are to promote and foster research and education in emergency and disaster medicine with particular interest in the application of new informatic technologies. Its ultimate goal is to improve health care system response in emergency, disaster and crisis situations through increasing the knowledge, skills and attitudes of health care providers based on scientific evidence and on field experience at regional, national and international levels.

CRIMEDIM is also a member of the established Emergency Medicine and Disaster Management Academy (EMDM Academy) based in Geneva^11^.

Rainbow for Africa Onlus is an Italian non-profit non-governmental organization established in 2009 whose mission is to foster quality and access to healthcare in Africa through sustainable interventions. Rainbow for Africa Onlus is primarily involved in development projects for the professional training of local healthcare personnel in Africa, and specifically focused on emergency and essential surgical care.

Rainbow for Africa Onlus has been working in Burkina Faso, Ethiopia, Senegal, Haiti and Sierra Leone. It is also involved in Continuous Medical Education courses for Italian healthcare personnel (Tropical Medicine and Ultrasound in low-resource settings) and public awareness campaigns, particularly in schools.

Both CRIMEDIM and Rainbow for Africa Onlus are members of the WHO Global Initiative for Emergency and Essential Surgical Care (WHO GIEESC)^12^.

## Methods

**Selection of Participants**

All residents enrolled in the Anesthesiology and Intensive Care residency program at the Università del Piemonte Orientale (Italy) were invited to participate in the pilot project. Residents were made aware that participation in the project was voluntary and would not affect their residency evaluation; however they were informed that participation to the online course was required before deployments. Candidates had to meet the following prerequisites:

- adequate level of professional competencies with regard to the medical activities to be performed as assessed by the director of the residency program;

- good knowledge of either English or French (B1 level of common European framework of reference for languages^16^ ) depending on the official language spoken in the host country;

- strong motivation for deployment evaluated by a questionnaire and interviews by representatives of the two institutions.

**Online Training**

The online training course was designed in July 2011 and hosted on the CRIMEDIM website using the Modular Object-Oriented Dynamic Learning Environment (MOODLE), a customisable, flexible and interactive Learning Management System (LMS)^17^ .

The selection of the topics were based on the review of existing professional organizations’ training programmes, definitive humanitarian and disaster medicine references, and the experience of the experts from the two institutions (including academic and government). All the experts were anaesthesiologists who had been deployed at least once in the aftermath of a major disaster or during a humanitarian aid program.

Following analysis of international guidelines on disaster response training, peer-reviewed publications^4^^,^ 6 ^,^ 13 ^,^ 14 ^,^ 17 , and programs such as the H.E.L.P. (Health Emergencies in Large Populations) and United Nations Disaster Management Training Programme (DMTP), learning objectives were fixed. Topics were than structured into a framework represented by subject headings. To identify the content details, a systematic literature search in Medline and Google Scholar with the key terms [training], [disaster], [crisis], [management], [humanitarian], [competency/ies] and [resource-poor and-constrained environments] was conducted. Complementary Internet searches were also performed, and resources from websites such as those from the World Health Organization (WHO), International Committee of Red Cross (ICRC) and Médecins Sans Frontières (MSF) reference guidelines were included. Curricular committee consensus on those entities regarded to be most important was required for item inclusion. The course was than constructed and resulted in 7 academic units with an estimated completion time of 32 hours (4 FTE – Full-Time Equivalent). A summary of the units is provided in Table 1.

In order to pass the online training, participants had to complete a final test to assess their level of knowledge. The test consisted of 12 multiple-choice questions on the topics dealt in the units of the training, such as:

- Primary evaluation of an unconscious trauma patient after a car accident
- Approach to a mass casualty incident due to fire in the hospital
- Management of a postpartum hemorrhage
- Evaluation of a burnt patient
- Techniques of loco-regional anesthesia and side effects of local anesthetics
- Ketamine anesthesia
- Anesthesia breathing circuits

**Table 1. Summary of units included in the online training course:** 

Unit	Description	Estimated Time (hours)
Introduction	The module provide definitions of Complex Emergencies definition (IASC, December 1994) and characteristics of UN response framework	1
Security in the field	The module included the "UN Basic Security in The Field" online training course^19^ and the report by ICRC on violence affecting healthcare personnel^20^ . For the module to be considered completed, each candidate had to submit the certificate provided upon completion of the "UN Basic Security in The Field" online training course.	5
Host Country: general information, health infrastructures and required vaccinations	The module provided relevant information about the host country profile, including historical context, demographic data, cultural context, economy and currency and health services and infrastructures. Candidates also had to comply with general rules as recommended by the Italian Ministry of Foreign Affairs' Crisis Unit^21^ and provide proof of having been given the required vaccinations.	2
Anesthesia at district hospital	The module discussed the main peculiar aspects of anesthesia at district hospital in a low-income country, and in particular: international standards for safe anesthesia^22^ , medicines available^23^ , available infrastructures and equipment^24^^,^ 25 , general and regional anesthesia techniques, including monitoring the anesthetized patient and postoperative care^26^ .	10
Surgery at district hospital	The module described the main features of surgical care at district hospital^27^^,^ 28 ^,^ 29 , with a focus on essential trauma care^30^ and management of burns.	6
Medical treatment in resource-constrained environment	The module covered the essentials of medical treatment in resource-constrained environment, including obstetrics in remote settings, according to MSF clinical guidelines^31^^,^ 32 .	5
Mental health and psychological support	The module presented mental health and psychological support in emergency settings according to IASC guidelines^33^	3

**Medical apprenticeships**

Upon completion of the aforementioned training course, candidates had the opportunity for a 30 day apprenticeship period designed in the framework of the field programs carried out by Rainbow for Africa in Burkina Faso, Senegal and Sierra Leone where safety and security were acceptable (i.e. no unstable environments). A senior anaesthesiologist with tutoring experience was present for the entire training period.

Grounding on the objectives of the development project, the needs of the host country, and the learning objectives of candidate’s curricula, an individual learning agreement was developed^13^ . The learning agreement recognized the student's abilities and included the educational objectives and the learning activities the student was expected to accomplish during the field practicum, as well as the components of the supervision which was agreed to and provided to the student.

During the apprenticeship, candidates had the opportunity of being actively involved in the clinical activities performed at the hospital they were assigned to, and specifically:

- anesthetic management of patients due to undergo surgical interventions (both scheduled and emergent),

- management of medical emergencies requiring life support maneuvers,

- postoperative medical care, and

- tutored outpatient case management.

Furthermore, candidates could take part in the training sessions organized for the local healthcare personnel. They were involved either in the planning prior to departure as programme designer or in the actual training sessions as instructor, or both. All planned activities were considered relevant to the candidate's residency curriculum. Framework agreements were then arranged between contributing parties and the local institutions; all candidates were given the required pre-deployment vaccinations as well as medical and professional insurance.

A debriefing was planned at the end of each apprenticeship with the purpose of evaluating learning outcomes. Though a standardized evaluation process was not used, such debriefing included a report on the activities performed and the related outcomes (phrased according to Bloom’s Taxonomy) written by the supervising senior anesthesiologist. Participants had the opportunity to further express their feedback to representatives of the involved organizations. Participants were also encouraged to provide suggestions on how to improve the program and were involved in course handovers between existing years of training.

**Self Evaluation**

At the end of the project participants were asked to complete a self-evaluation questionnaire with the aim of analyzing the impact of the project on their professional skills, personal perspective of the value of the project and to document their opinions about the value of the professionalization of the humanitarian aid sector. Participants expressed their level of agreement to 10 questions using a five-point Likert-type scale response (Likert items: strongly disagree; disagree; don’t agree nor disagree; agree; strongly agree). Participants were allowed to further comment on the project using an open-ended item questionnaire. The questionnaire was administered using SurveyMonkey (SurveyMonkey LLC, Palo Alto, California USA) via a direct e-mail invitation to complete the survey.

Written informed consent was obtained from each participant prior to the start of the course. To assure confidentiality, the students submitted the satisfaction questionnaire anonymously. Since all data were de-identified, the evaluation was deemed exempt from institutional review approval by the local Ethics Committee.

## Results

Currently the training course has been successfully completed by 8 Anesthesia and Intensive Care Medicine residents (Table 2) who voluntary participated in the program, met prerequisites, proficiently passed the online training final test (defined as having correctly answered to at least 66% of the test questions) and therefore had the opportunity to participate in a medical apprenticeship described as follows (Table 3):

- Teams of residents accounting for a total of 6 participants joined the development program carried on in Burkina Faso (October 2011, March 2012, September 2012). Under tutorage of a senior anesthesiologist

## References

[1] ALNAP. The State of the Humanitarian System 2012. Available at http://www.alnap.org/resource/6565. Last accessed on 01/27/2014.

[2] United Nations Humanitarian Response Review 2005. Available at http://www.humanitarianinfo.org/iasc/downloadDoc.aspx?docID=2969&type=pdf. Last accessed on 08/12/2013.

[3] Global Health Cluster Concept Paper: Coordination and registration of providers of foreign medical teams in the humanitarian response to sudden-onset disasters. Available at http://www.who.int/hac/global_health_cluster/about/policy_strategy/fmt_concept_paper_27_May.pdf. Last accessed on 08/12/2013.

[4] Walker P, Hein K, Russ C, Bertleff G, Caspersz D. A blueprint for professionalizing humanitarian assistance. Health Aff (Millwood). 2010 Dec;29(12):2223-30. PubMed PMID:21134923. 21134923 10.1377/hlthaff.2010.1023

[5] Kene M, Pack ME, Greenough PG, Burkle FM Jr. The professionalization of humanitarian health assistance: report of a survey on what humanitarian health workers tell us. Prehosp Disaster Med. 2009 Jul-Aug;24 Suppl 2:s210-6. PubMed PMID:19806543. 19806543 10.1017/s1049023x00021610

[6] CBHA Core Humanitarian Competencies Framework. Available at http://www.thecbha.org/media/website/file/Competencies_Framework_2012_colour.pdf. Last accessed on 08/12/2013.

[7] UK-DFID Humanitarian Emergency Response Review 2011. Available at https://www.gov.uk/government/uploads/system/uploads/attachment_data/file/67579/HERR.pdf. Last accessed on 08/12/2013.

[8] Jayaraman SP, Ayzengart AL, Goetz LH, Ozgediz D, Farmer DL. Global health in general surgery residency: a national survey. J Am Coll Surg. 2009 Mar;208(3):426-33. PubMed PMID:19318005. 19318005 10.1016/j.jamcollsurg.2008.11.014

[9] https://crimedim.med.unipmn.it/ - CRIMEDIM official website. Last accessed on 05/15/2014

[10] http://www.rainbow4africa.org - Rainbow for Africa official website. Last accessed on 05/15/2014

[11] http://www.emdmacademy.org/Members/ - EMDM academy official website. Last accessed on 05/15/2014

[12] WHO Global Initiative for Essential and Emergency Surgical Care. http://www.who.int/surgery/globalinitiative/en/index.html

[13] McDermott MM, Curry RH, Stille FC, Martin GJ. Use of learning contracts in an office-based primary care clerkship. Med Educ. 1999 May;33(5):374-81. PubMed PMID:10336774. 10336774 10.1046/j.1365-2923.1999.00346.x

[14] Pan American Health organization, World Health Organization. Proceedings of the WHO/PAHO Technical consultation on International Foreign Medical Teams (FMTs) post Sudden Onset Disasters (SODs), December 7-9, 2010. Havana, Cuba. http://new.paho.org/disasters/index. php?option=com_docman&task=doc_download&gid=1761&Itemid=. Last accessed on 01/26/2012

[15] Drain PK, Primack A, Hunt DD, Fawzi WW, Holmes KK, Gardner P. Global health in medical education: a call for more training and opportunities. Acad Med. 2007 Mar;82(3):226-30. PubMed PMID:17327707. 17327707 10.1097/ACM.0b013e3180305cf9

[16] Common European framework of reference for languages. Available at http://europass.cedefop.europa.eu/en/resources/european-language-levels-cefr. Last accessed on 08/14/2013.

[17] https://moodle.org/

[18] Rössler B, Marhofer P, Hüpfl M, Peterhans B, Schebesta K. Preparedness of anesthesiologists working in humanitarian disasters. Disaster Med Public Health Prep. 2013 Aug;7(4):408-12. PubMed PMID:24229525. 24229525 10.1017/dmp.2013.40

[19] UN Basic Security in the field training course. Available at https://training.dss.un.org/‎. Last accessed on 08/15/2013.

[20] ICRC Health care in danger: a sixteen-country study. Available at http://www.icrc.org/eng/resources/documents/report/hcid-report-2011-08-10.htm. Last accessed on 08/15/2013.

[21] Website from the Italian Ministry of Foreign Affairs about recommendations when travelling worldwide Available at http://www.viaggiaresicuri.it/index.php?id=62. Last accessed on 08/15/2013 [website in Italian].

[22] WFSA International Standards for a Safe Practice of Anaesthesia 2010. Available at http://www.anaesthesiologists.org/guidelines/practice/2008-international-standards-for-a-safe-practice-of-anaesthesia. Last accessed on 08/14/2013.

[23] WHO Model Lists of Essential Medicines. Available at http://www.who.int/medicines/publications/essentialmedicines/en/index.html. Last accessed on 08/15/2013.

[24] WHO Generic Essential Emergency Equipment List. Available at http://www.who.int/surgery/publications/EEEGenericListFormatted%2006.pdf. Last accessed on 08/14/2013.

[25] WHO Guide to Anesthetic Infrastructure and Supplies at various levels of Health Care Facilities Available at http://www.who.int/surgery/publications/GuideAnestheticInfrastFormatted06.pdf. Last accessed on 08/14/2013.

[26] WHO Anesthesia at the district hospital manual. Available at http://whqlibdoc.who.int/publications/9241545275.pdf. Last accessed on 08/14/2013.

[27] WHO Surgical Care at the district hospital manual. Available at http://www.who.int/surgery/publications/en/SCDH.pdf. Last accessed on 08/14/2013.

[28] Chackungal S, Nickerson JW, Knowlton LM, Black L, Burkle FM, Casey K, Crandell D, Demey D, Di Giacomo L, Dohlman L, Goldstein J, Gosney JE Jr, Ikeda K, Linden A, Mullaly CM, O'Connell C, Redmond AD, Richards A, Rufsvold R, Santos AL, Skelton T, McQueen K. Best practice guidelines on surgical response in disasters and humanitarian emergencies: report of the 2011 Humanitarian Action Summit Working Group on Surgical Issues within the Humanitarian Space. Prehosp Disaster Med. 2011 Dec;26(6):429-37. PubMed PMID:22475370. 22475370 10.1017/S1049023X12000064

[29] WHO Emergency Triage Assessment and Treatment manual. Available at http://whqlibdoc.who.int/publications/2005/9241546875_eng.pdf. Last accessed on 08/14/2013.

[30] WHO Guidelines for essential trauma care. Available at http://www.who.int/violence_injury_prevention/publications/services/guidelines_traumacare/en/. Last accessed on 08/15/2013.

[31] MSF Clinical guidelines - diagnosis and treatment manual. Latest edition available at http://refbooks.msf.org/msf_docs/en/clinical_guide/cg_en.pdf. Last accessed on 08/14/2013.

[32] MSF Obstetrics in remote settings manual. Available at http://refbooks.msf.org/msf_docs/en/obstetrics/obstetrics_en.pdf. Last accessed on 08/15/2013.

[33] IASC. Mental Health and Psychosocial Support in Emergency Settings: What should Humanitarian Health Actors know?. Available from IASC website http://www.humanitarianinfo.org/iasc/.

